# Fibromatoses of Head and Neck: Case Series and Literature Review

**DOI:** 10.5041/RMMJ.10444

**Published:** 2021-07-20

**Authors:** Muddasir Bhati, Gurukeerthi Balakrishna, Kamaldeep Joshi, Kajari Bhattacharya, Munita Bal, Sarbani Ghosh Laskar, Shwetabh Sinha, Amit Joshi, Poonam Joshi, Sudhir Nair, Pankaj Chaturvedi

**Affiliations:** 1 Department of Head and Neck Surgery, Advanced Centre for Treatment, Research and Education in Cancer, Tata Memorial Hospital, Homi Bhabha National Institute, Mumbai, India; 2 Department of Radiodiagnosis and Imaging, Advanced Centre for Treatment, Research and Education in Cancer, Tata Memorial Hospital, Homi Bhabha National Institute, Mumbai, India; 3 Department of Pathology, Advanced Centre for Treatment, Research and Education in Cancer, Tata Memorial Hospital, Homi Bhabha National Institute, Mumbai, India; 4 Department of Radiotherapy, Advanced Centre for Treatment, Research and Education in Cancer, Tata Memorial Hospital, Homi Bhabha National Institute, Mumbai, India; 5 Department of Medical Oncology, Advanced Centre for Treatment, Research and Education in Cancer, Tata Memorial Hospital, Homi Bhabha National Institute, Mumbai, India

**Keywords:** Aggressive fibromatosis, desmoid tumors, fibromatosis, rare tumors

## Abstract

**Objective:**

The objective of this study was to retrospectively review clinical data, management protocols, and clinical outcomes of patients with fibromatoses of head and neck region treated at our tertiary care center.

**Methods:**

We retrospectively reviewed the medical records of 11 patients with confirmed histopathological diagnosis of fibromatosis registered in the Department of Head and Neck Surgery at Tata Memorial Centre, India, between 2009 and 2019. Various clinical and pathological features and treatment modalities were evaluated.

**Results:**

Age at diagnosis ranged between 18 and 74 years, with a median age of 36 years. The female-to-male ratio was 5:6. Supraclavicular fossa (*n*=4) was the most common subsite of origin in the neck (*n*=8). The lateral (*n*=2) and posterior cervical regions (*n*=2) were other common neck subsites. Less commonly involved sites were the mandible (*n*=1), maxilla (*n*=1), and thyroid (*n*=1). A total of eight patients underwent surgery at other centers before being referred to us for further management. Out of a total 11 patients, nine patients had unresectable disease at presentation. Six of the patients with unresectable disease received a combination of weekly doses of vinblastine 6 mg/m^2^ and methotrexate 30 mg/m^2^ for a median duration of 6 months (range 6–18 months) followed by hormonal therapy with tamoxifen. Three patients received metronomic chemotherapy followed by hormonal therapy. One treatment-naive patient with fibromatosis of posterior cervical (suboccipital) region underwent R2 resection (excision of bulk of the tumor with preservation of critical structures) at our center along with adjuvant radiotherapy. One pregnant patient reported to us after undergoing surgery outside and defaulting radiotherapy. During median follow-up of 29 months (range 1–77 months), six patients had stable disease, and four patients had disease reduction. Disease progression was seen in one patient. The two-year progression-free survival (PFS) was 90% (95% CI 70%–100%).

**Conclusion:**

Gross residual resection (R2) was the mainstay of surgical treatment in our series, as obtaining clear surgical margins is seldom possible in these locally aggressive tumors. Radiotherapy, chemotherapy, and hormonal therapy are the other preferred and more conservative treatment modalities. The goal of surgery should be preserving function with minimal or no morbidity. As fibromatoses in the head and neck region are extremely rare, their treatment awaits the development of standard treatment protocols.

## INTRODUCTION

Fibromatoses are a heterogeneous group of rare soft tissue tumors, characterized by their infiltrative growth and origin from fibroblasts or myofibroblasts. Although lacking malignant and metastatic potential, they are locally aggressive by nature and tend to recur, often making patient management challenging. Fibromatoses account for 0.03% of all neoplasms and 3% of soft tissue tumors.^1^ Among the various categories of fibromatoses, extra-abdominal desmoid tumors constitute the largest subgroup, 12% of which arise from the head and neck region.^2^ Fibromatoses originating in the head and neck region are characterized by aggressive behavior and a high local recurrence rate of 20%–80% following resection.^3^,^4^ In head and neck fibromatoses, the propensity to cause massive destruction and erosion of adjacent bones, infiltration of critical structures, and functional compromise requires a comprehensive and reliable treatment strategy. This article shares our institution’s experience in management of rare fibromatoses of the head and neck region.

## METHODOLOGY

This retrospective study was performed in Tata Memorial Hospital, Mumbai, India. The medical records of 540 registered patients from 2009 to 2020 were retrieved and critically reviewed to identify all patients who had been registered in our department of head and neck surgery with a histologically proven diagnosis of fibromatoses. The demographic and clinical details such as site, symptoms, treatment, and outcome were obtained and analyzed.

## RESULTS

**Figure 1:**
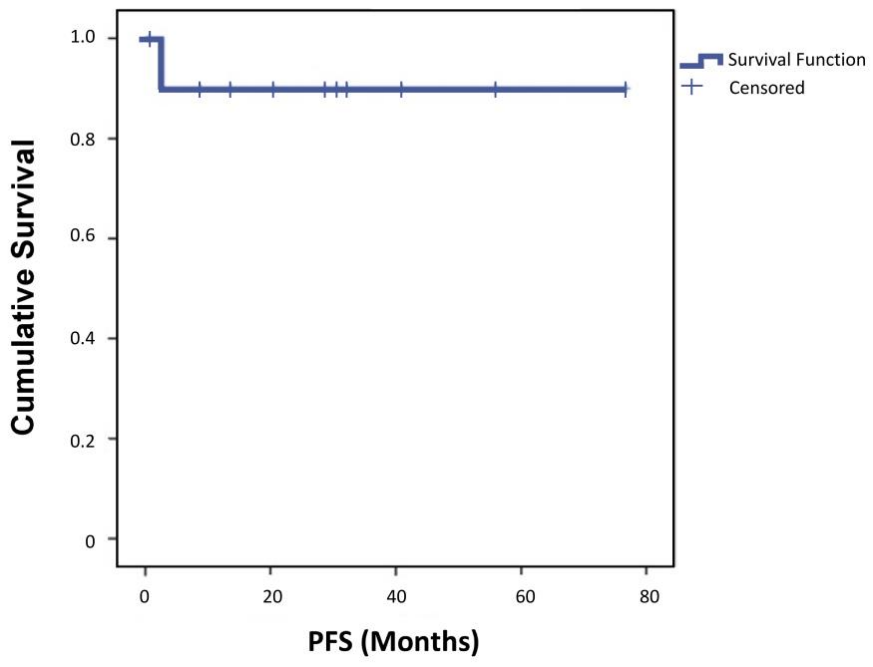
Two-year Progression-free Survival (PFS)=90% (95% CI 70%–100%). The survival function gives the probability that a patient will survive beyond any specified time. CI, confidence interval.

Eleven patients with fibromatoses were managed in the Head and Neck Unit at our center between 2009 and 2020. The median age at diagnosis was 36 years, with range being 18–74 years. The most common site was cervical (neck) region (*n*=8). Less commonly involved sites were mandible (*n*=1), maxilla (*n*=1), and thyroid (*n*=1). Out of the 11 patients, six were asymptomatic at presentation. In those who were symptomatic (*n*=5), altered sensation and pain were the most common symptoms. At the end of follow-up, none of the symptomatic patients had complete resolution of symptoms. Gross residual resection (R2) had been performed on all patients who had undergone surgery (*n*=8), seven at other centers and one at our center. The latter patient underwent R2 for an unresectable tumor due to mass infiltration into the paraspinal muscles. Among the patients who underwent surgery, six patients received combination of weekly doses of vinblastine 6 mg/m^2^ and methotrexate 30 mg/m^2^ for a median duration of 6 months (6–18 months) followed by hormonal therapy with tamoxifen. A total of two patients received metronomic chemotherapy followed by hormonal therapy, and one patient received chemotherapy followed by hormonal therapy. One patient received radiotherapy (a total of 60 Gy in 30 2-Gy fractions) starting on day 34 post R2. Stable disease was achieved in a total of six patients during follow-up. Disease regression was noticed in four patients. Disease progression was noticed in one patient having fibromatosis of the maxilla; the patient underwent surgery with adjuvant chemo-radiotherapy before being treated with metronomic chemotherapy at our center. The clinical details of the cases are given in Table 1. During the median follow-up period of 29 months (range 1–77 months), the two-year progression-free survival (PFS) was 90% (95% confidence interval [CI] 70%–100%) (Figure 1).

**Table 1:** Clinical Details of Head and Neck Fibromatosis Study Group.

Patient # / Site	Symptoms	Structures Involved	Treatment Modality	PresentStatus	Follow-up (Months)
#1 / Neck	Pain, left neck swelling, left upper limb motor deficit	Left paravertebral C4–C7 levels	Surgery + chemotherapy + hormonal therapy	Stable disease	77
#2 / Neck, supraclavicular fossa	Swelling over left supraclavicular fossa	Left brachial plexus with encasement of vertebral artery	Surgery + chemotherapy + hormonal therapy	Stable disease	32
#3 / Mandible	Swelling below right lower jaw	Right lower gingivo-buccal sulcus, floor of mouth, involving strap muscles but not the thyroid cartilage	Surgery + chemotherapy + hormonal therapy	Disease regression	31

#4 / Neck	Right hand swelling, tingling and numbness	Lesion extending from C4–D2 and extension into C5–C6 nerve roots encasing subclavian artery	Metronomic chemotherapy	Stable disease	1*
#5 / Neck, supraclavicular fossa	Swelling over left supraclavicular fossa	Loss of fat planes in superior trunk of brachial plexus	Metronomic chemotherapy	Stable disease	56

#6 / Posterior neck	Swelling in left posterior side of neck	Left posterior spine region	Surgery + metronomic chemotherapy + hormonal therapy	Disease regression	41

#7 / Posterior neck	Swelling in posterior side of neck	Nape of neck, vertebral artery, paraspinal muscles	Surgery + adjuvant radiotherapy	Disease regression	29

#8 / Thyroid	Swelling, change in voice, difficulty swallowing	Large lobulated mass in retropharyngeal region, tracheal compression and displacement	Surgery + radiotherapy + chemotherapy + hormonal therapy	Disease regression	21
#9 / Maxilla	Pain, swelling over left side of face, vision loss, ear discharge, nasal blockage	Intracranial extension	Surgery + radiotherapy + chemotherapy + hormonal therapy	Disease progression	4

#10 / Left supraclavicular fossa	Swelling in left supraclavicular region, left shoulder pain radiating to left arm	Left supraclavicular fossa lesion with loss of fat planes in relation to left common carotid artery and subclavian artery	Chemotherapy + hormonal therapy	Stable disease	14

#11 / Left supraclavicular fossa	Swelling in left supraclavicular region	Brachial plexus lesion encasing the common carotid artery	Surgery + radiotherapy (defaulted) + metronomic chemotherapy	Stable disease	9

* This patient experienced neck swelling for 7 years, which was progressive in nature. She presented with compressive symptoms but was inoperable. The patient received metronomic chemotherapy for 1 month but was lost to follow-up after that.

## DISCUSSION

**Figure 2:**
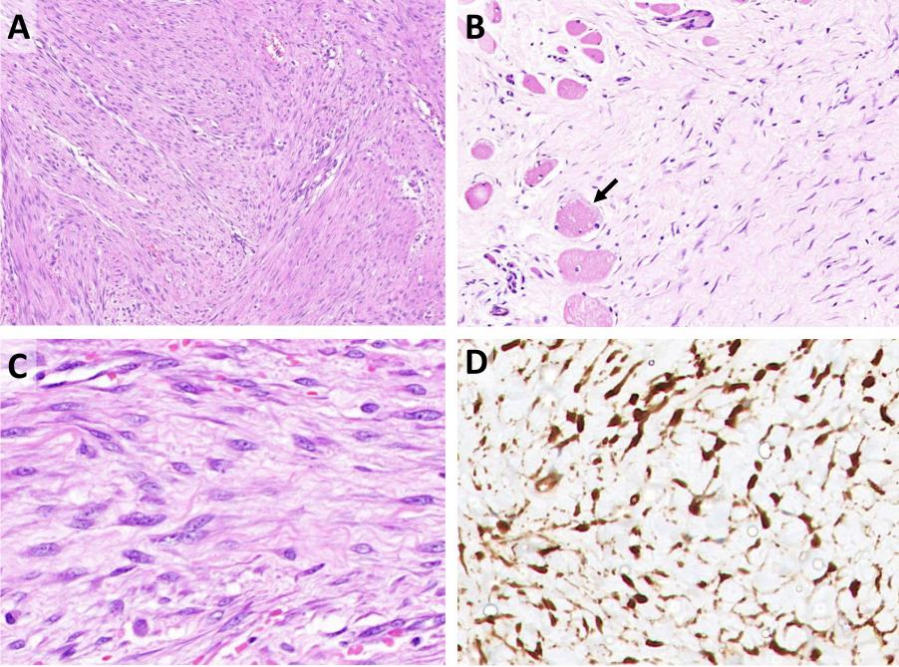
Histopathology of Fibromatosis Cases. **A:** Low magnification shows paucicellular spindle cell proliferation composed of fibroblastic cells embedded in a collagenous stroma. **B:** Fibroblastic proliferation infiltrating skeletal muscle cells (black arrow). **C:** Higher magnification shows spindle cells with elongated nuclei, pale nuclear chromatin, and inconspicuous nucleoli in a collagenous stroma. **D:** Spindle cells show strong and diffuse nuclear beta-catenin.

Fibromatoses are rare neoplastic disorders of fibroblastic origin with unknown etiology. Few of these tumors have a syndromic association. The incidence of desmoid tumors in the general population is 2–4 per million population per year.^1^ Head and neck fibromatoses worldwide constitute an estimated 10%–15% of all the fibromatoses occurring in all parts of the body.^5^ Even though the etiology is unknown, many authors believe that trauma or prior surgeries may stimulate the development of these tumors.^5^,^6^ Histologically, the tumor is composed of mature fibroblasts arranged in spindle form with a tendency to clump in scattered foci, and cellularity varies from region to region. The features suggestive of malignancy such as hyperchromasia, mitotic figures, and anaplasia are not seen in these lesions.^7^ The various histological features are shown in Figure 2.

**Figure 3:**
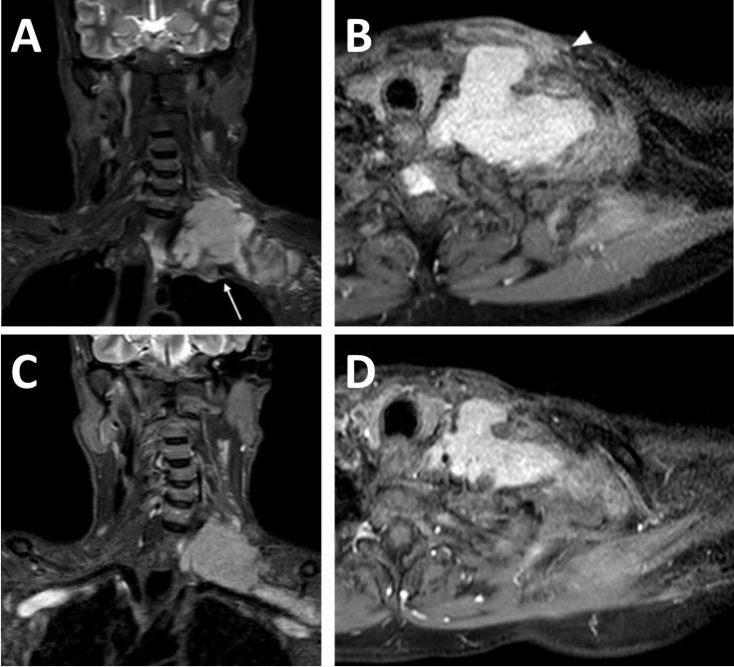
Coronal STIR and Axial Post Contrast MR Images of Neck and Supraclavicular Region Revealing Supraclavicular Swelling in a 55-year-old Patient. A and B: Infiltrative left brachial plexus lesion indenting on lung apex (arrow, A), encasing cervical vessels, with soft tissue edema and enhancement extending up to overlying skin (arrowhead, B) shown on pre-chemotherapy coronal short tau inversion recovery (STIR) (A) and axial post contrast T1-weighted imaging (B). C and D: Corresponding post-chemotherapy images show significant reduction in size of the lesion.

**Figure 4:**
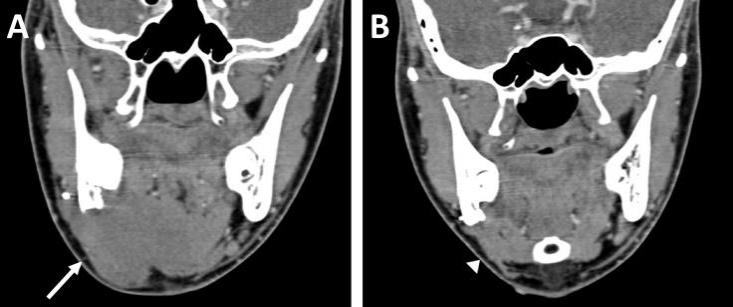
Hard Swelling in the Right Submandibular Region of an 18-year-old Patient. **A:** Pre-chemotherapy CT in coronal reconstruction shows homogeneous mildly enhancing lesion in the right submandibular region and floor of mouth (arrow, **A**). **B:** Post-chemotherapy CT shows significant decrease in lesion size (arrowhead, **B**).

**Figure 5:**
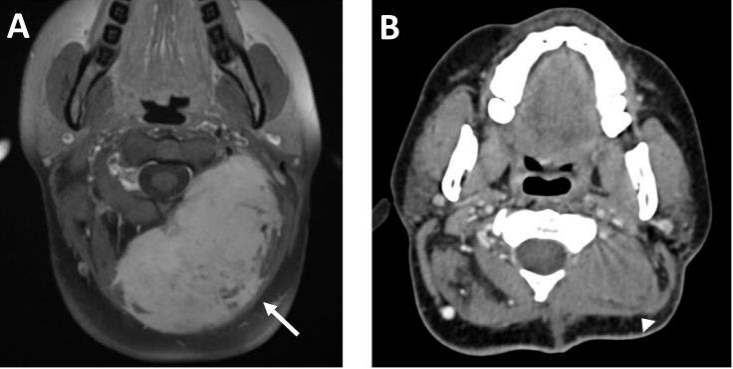
Swelling in the Left Posterior Neck Region of a 42-year-old Female Patient for Two Years. **A:** Pre-surgery, axial post contrast MRI of neck shows heterogeneously enhancing solid lesion reaching up to the C1 vertebra abutting the spinal epidural space on left (arrow, **A**). **B:** Post surgery and radiotherapy, axial CT showing significant decrease in the size of the lesion (arrowhead, **B**).

Imaging has an important role in the preoperative assessment of disease extent and its relation to vital structures including great vessels of neck, skull base, and brachial plexus. Although a computed tomography (CT) scan can detect the size and extent of the tumor precisely, magnetic resonance imaging (MRI) is preferred as it has better sensitivity in delineating the soft tissue involvement. The CT scan may complement MRI in order to rule out bony erosion in selected cases. In our study, pre-treatment imaging that included CT scan, MRI, or both was performed for all patients. Comparisons of pre-treatment and post-treatment radiological images in our study are shown in Figures 3, 4, and 5.

Gross residual resection is still considered as the mainstay of surgical treatment for fibromatoses. Radical or complete resection of this tumor is seldom possible due to the proximity of vital structures. Incomplete clearance is accompanied by a high rate of local failure. The recurrence rate can be as high as 82%–86% with positive margins, as compared to 22%–25% with negative margins.^8^,^9^ Wang et al. have reported long-term disease-free survival even after marginal or intra-lesional excision.^5^ Reitamo et al. found little difference in recurrence rate after complete and incomplete resections, i.e. 17% and 25%, respectively.^1^ Plukker et al., with their experience in managing 39 cases of aggressive fibromatoses, recommend a margin of at least 3 cm as adequate for resection, with good loco-regional control of 59% during their median follow-up period of 72 months. They achieved complete resection in only 32 patients, and 5 patients underwent incomplete resection with less than 1 cm and received post-operative radiotherapy.^4^ Radiotherapy is an established modality of treatment for residual tumor following resection or after recurrence. In selected cases adjuvant external beam radiotherapy or interstitial brachytherapy can be an alternative treatment option to avoid mutilating surgeries and serious consequences.^10^ In the study by Plukker et al., local control was obtained in 13 of 14 patients who received radiotherapy with doses of more than 50 Gy over a period of 5 weeks after marginal or incomplete resection of primary or recurrent lesions.^4^ However, Hoos et al. could not demonstrate benefits of adjuvant radiation in their case series of 29 patients with head and neck desmoid tumor which were treated primarily by surgery, out of which 11 patients were irradiated.^6^ In our study, significant disease regression was noticed in a patient treated with surgery and radiotherapy (Figure 3). A multicenter cohort study was initiated by the German Cooperative Group on Radiotherapy for Benign Diseases (GCG-BD). Long-term data were obtained for 345 patients with aggressive fibromatoses from all anatomical sites. Only 17 cases with fibromatoses from the head and neck region were found, and all of them received radiotherapy alone. The 5-year loco-regional control rate was 81.5% after primary radiotherapy for unresectable tumors and 79.6% after postoperative radiotherapy for resectable disease.^11^ However, the site-specific control rate was not analyzed. The study concluded that radiotherapy can be used in cases of incomplete resection or unresectable tumors. However, caution is warranted in younger patients due to the well-known effects of radiation on impairing bone growth and the potential for delayed development of secondary cancers. For this reason, tamoxifen was added as an alternative to radiotherapy in a child with a positive margin after the resection of a sinonasal lesion, and the results were promising.^12^ Since most of the patients treated at our center had undergone R2 elsewhere, they received adjuvant therapy in the form of radiotherapy, chemotherapy, and/or hormonal therapy. In our study, only three patients received adjuvant radiotherapy. While one of them experienced disease regression and another had stable disease, the patient with maxilla fibromatosis experienced disease progression. Hence, due to the limited number of patients receiving adjuvant radiotherapy, we cannot comment upon its role in achieving local control after gross residual resection.

Even though desmoid tumors are categorized as benign, they do respond to chemotherapy which provides symptomatic relief in the majority of patients. In a trial of 30 patients, weekly vinblastine and methotrexate resulted in a partial response rate of 40% and disease stability in 60% of patients. The 10-year progression-free interval was 67% at a median follow-up of 75 months.^13^ Skapek et al. used a similar regimen in 26 children with aggressive fibromatoses; four patients had partial response, three had a minor response, and one had complete response.^14^ These findings are similar to our study where six patients had stable disease and four patients had disease regression. In 1944, Lipschütz et al. reported the use of progesterone to inhibit the initiation of fibromatoses in guinea pigs when exposed to high doses of estrogen, although this finding could not be reproduced in other animals.^15^ Hansmann used intramuscular progesterone to treat fibromatosis patients with a favorable result. Cyclooxygenase seems to play a key role in the pathogenesis of desmoid tumors. Administration of non-steroidal anti-inflammatory drugs (NSAIDs) and tamoxifen seems to be the most effective non-cytotoxic drug treatment in adults and has low toxicity. In a series of 25 patients treated with high-dose tamoxifen and sulindac, the efficacy of this regime was demonstrated.^16^ In one series, patients who received tamoxifen with NSAIDs had the greatest response when compared to other agents (NSAID with warfarin and vitamin K1); out of 7 patients, 5 had a major reduction in tumor size.^17^ Many studies do not report on the existence of estrogen receptors in the tumor, making it difficult to conclude the efficacy of antiestrogen agents. Hence the role of adjuvant hormonal treatment is equivocal.^18^

In our series, nine out of 11 patients received tamoxifen following definitive treatment. Among them, six patients were asymptomatic and five had disease with mild symptoms. Most recently, the activity of the tyrosine kinase inhibitor, sorafenib, against progressive desmoid tumors was reported in a series of 26 patients. The symptoms in the majority (70%) of patients significantly improved, as seen in the 17 patients with resulting stable disease and the six with partial response.^19^ In our series, we observed satisfactory disease control with the use of chemotherapeutic agents. Since surgery is contraindicated in patients with a poor score on the Eastern Cooperative Oncology Group (ECOG) scale, as well as in patients at risk of permanent damage to vital structures, these patient groups should be treated upfront with other modalities.

## CONCLUSION

Head and neck fibromatosis is a rare condition with heterogeneity in presentation, proximity to vital structures, and locally aggressive nature. These features make its treatment extremely challenging. Because of its rarity, variability in behavior, and the characteristics of these tumors, a standard treatment protocol has not yet been established. Although retrospective in nature, the current study adds further insight into various aspects of management of this rare entity. This study has inherent limitations as it was retrospective, with a limited number of patients. However, as per our experience, we could conclude that surgery followed by multimodality management offers the best control, if not cure, for fibromatosis of head and neck region.

## Abbreviations

CI: confidence interval
CT: computed tomography
MRI: magnetic resonance imaging
PFS: progression-free survival
R2: gross residual resection
